# Proteolysis Targeting Chimera Agents (PROTACs): New Hope for Overcoming the Resistance Mechanisms in Oncogene-Addicted Non-Small Cell Lung Cancer

**DOI:** 10.3390/ijms252011214

**Published:** 2024-10-18

**Authors:** Nicoletta Cordani, Daniele Nova, Luca Sala, Maria Ida Abbate, Francesca Colonese, Diego Luigi Cortinovis, Stefania Canova

**Affiliations:** 1 School of Medicine and Surgery, University of Milano-Bicocca, 20900 Monza, Italy; diego.cortinovis@unimib.it; 2 Medical Oncology Unit, Fondazione Istituti di Ricovero e Cura a Carattere Scientifico (IRCCS) San Gerardo dei Tintori, 20900 Monza, Italy; daniele.nova@irccs-sangerardo.it (D.N.); luca.sala@irccs-sangerardo.it (L.S.); mariaida.abbate@irccs-sangerardo.it (M.I.A.); francesca.colonese@irccs-sangerardo.it (F.C.)

**Keywords:** PROTAC, oncogene-addicted NSCLC, TKI resistance

## Abstract

Non-small cell lung cancer (NSCLC) remains a disease with a poor prognosis despite the advances in therapies. NSCLC with actionable oncogenic alterations represent a subgroup of diseases for which tyrosine kinase inhibitors (TKIs) have shown relevant and robust impact on prognosis, both in early and advanced stages. While the introduction of powerful TKIs increases the ratio of potentially curable patients, the disease does develop resistance over time through either secondary mutations or bypass activating tracks. Therefore, new treatment strategies are being developed to either overcome this inevitable resistance or to prevent it, and proteolysis targeting chimera agents (PROTACs) are among them. They consist of two linked molecules that bind to a target protein and an E3 ubiquitin ligase that causes ubiquitination and degradation of proteins of interest. In this paper, we review the rationale for PROTAC therapy and the current development of PROTACs for oncogene-addicted lung cancer. Moreover, we critically analyze the strengths and limitations of this promising technique that may help pave the way for future perspectives.

## 1. Introduction

Non-small cell lung cancer (NSCLC) ranks consistently among the most frequently diagnosed cancers and is one of the leading causes of death globally [1]. However, the landscape has begun to change exponentially in the past decade, and the diagnostic and therapeutic standards for this disease have completely changed. The continuous technological development has increased our understanding of cancer cell biology, enabling the molecular characterization of the disease and the development of targeted drugs based on the tumour’s specific patterns. Thus, molecular testing is mandatory from the diagnosis, especially in adenocarcinoma, using next-generation sequencing (NGS) technology to identify subgroups of NSCLC with targetable driver oncogenic mutations.

With the advent of targeted molecular therapy, the prognosis and the quality of life of NSCLC patients have improved compared with those of patients treated with chemotherapy. However, the development of resistance to targeted treatments poses a significant limitation to their long-term efficacy, and the prognosis for these patients with advanced-stage disease, although improved, remains poor [2]. Thus, the onset of resistance generates significant challenges in the treatment process and requires continuous research for innovative and alternative strategies.

*EGFR* is the most frequent driver in NSCLC, accounting for about 10–15% of adenocarcinoma. The most common mutations are *EGFR* Ex19Del and *EGFR* L858R [3,4], for which the FLAURA study demonstrated that osimertinib, a third-generation tyrosine kinase inhibitor (TKI), is currently the best option in the first line in terms of progression free survival (PFS) and overall survival (OS) [5,6].

Osimertinib has also managed to overcome the resistance related to the emergence of the T790M mutation, which causes the refractoriness of treatment with earlier-generation TKIs [7]. However, even for osimertinib, several resistance mechanisms have been described that can counteract its effectiveness [8,9,10]. Innovative approaches to prevent them include escalation strategies such as the combination of osimertinib and chemotherapy, applied in the FLAURA2 trial and the association of amivantamab, an EGFR-MET bispecific antibody, and lazertinib, a third-generation TKI, in previously untreated EGFR positive NSCLC patients as presented in the MARIPOSA trial [11,12].

The ALK receptor tyrosine kinase can present translocations that occur in about 5% of patients with NSCLC, the most common being EML4:ALK [13,14]. Crizotinib was the first targeted drug developed against this driver, but secondary resistance mutations emerged that limited its effectiveness in controlling the disease [15]. Alectinib, a second-generation TKI, and lorlatinib, a third-generation TKI, showed an improvement over crizotinib in terms of response rates as well as PFS, but, once again, resistance may emerge [16,17,18,19].

Similarly, *ROS1, BRAF, RET, MET, KRAS, HER2*, and *NTRK* are actionable drivers against which powerful TKIs have been developed, but the disease develops resistance over time. Currently, the treatment of oncogene-addicted NSCLC following progression to targeted therapies mostly includes chemotherapy, as immunotherapy has shown low efficacy in these subgroups.

Therefore, the loss of treatment efficacy due to these resistance mechanisms and the subsequent disease progression underlines the need and necessity to continue research towards developing new therapies. In this scenario, proteolysis targeting chimera agents (PROTACs) comes into play.

## 2. PROTAC: Structure and Mechanism of Actions

The ubiquitin-proteasome system (UPS) is crucial for maintaining cellular protein balance. It includes chaperones, which corrects protein misfolding, and the 26S Proteasome, which removes damaged proteins to ensure a healthy cell environment [20,21,22,23]. The proteasome-mediated degradation of proteins requires the covalent attachment of ubiquitin (Ub) to the substrate. This process involves the sequential action of three enzymes, namely the E1 ubiquitin-activating enzyme, the E2 ubiquitin-conjugating enzyme, and the E3 ubiquitin ligase. The E1 enzyme, also known as the ubiquitin-activating enzyme 1 (Uba1), plays an essential role in initiating this cascade. It activates Ub by forming an ATP-dependent complex, referred to as the E1-Ub conjugate. This complex then transfers Ub to the E2 enzyme. Subsequently, Ub is transferred from the E1 to the E2 enzyme via a trans-thioesterification reaction. In the final step, the E3 Ub ligase simultaneously binds both E2-Ub and the substrate, facilitating the ultimate transfer of Ub, either directly from the E2 to the substrate or indirectly via the E3 Ub ligase itself, depending on the E3 ligase family. Substrates tagged with ubiquitin (Ub) are destined for the 26S proteasome for degradation [24].

**Figure 1:**
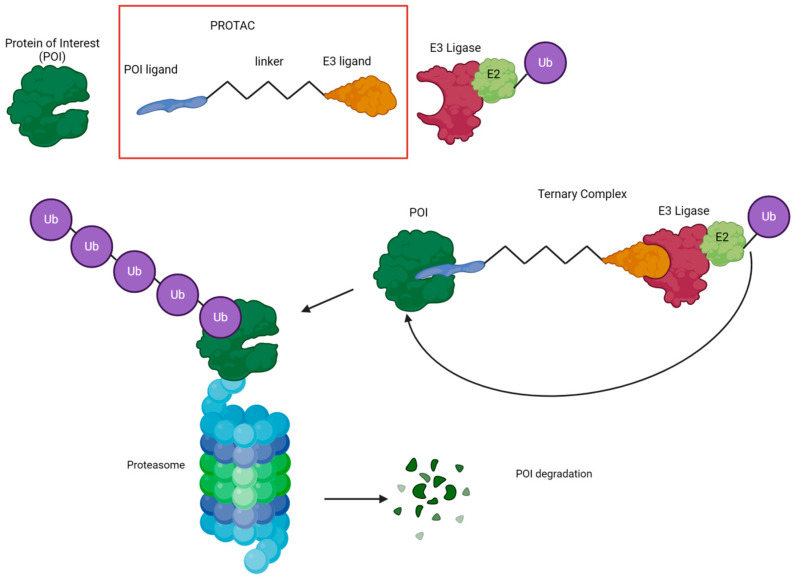
Proteolysis targeting chimeras (PROTACs). Schematic model and mechanism of action. Description of the schematic structure of PROTAC and the mechanism of degrading the protein of interest via ubiquitination and proteasome-mediated degradation.

PROteolysis TArgeting Chimera (PROTAC) is a novel technology that utilizes the UPS to degrade a protein of interest (POI) (Figure 1). This technology was first described in 2001 by Sakamoto et al. [25]. He described the use of bifunctional molecules as a means to recruit a ubiquitin ligase to a target of interest for protein degradation. Subsequently, Sakamoto and colleagues demonstrated that estrogen receptors and androgen receptors are downregulated in vitro in human cells treated with PROTACs [26]. Their approach increases protein turnover in the cell, suggesting that their methodology can be applied to proteins involved in human diseases. In 2008, the first entirely small-molecule PROTAC was described [27]. The process involved linking a small molecule ligand for the E3 ubiquitin ligase MDM2 to an androgen receptor ligand using a linker. This led to the degradation of the androgen receptor in cells.

PROTACs have a modular design consisting of an E3 moiety, a target moiety, and a linker, which sets them apart from monovalent molecular degraders. This approach also presents challenges, especially in terms of the chemical properties required for drug development [27,28]. Due to the large size of PROTACs (≥700 Da), extensive empirical investigation is required to achieve suitable pharmacokinetic properties for clinical use. Bondeson et al. demonstrates that efficient degradation can occur using both shorter (PROTAC_ERRa) and longer (PROTAC_RIPK2) linkers. However, the effectiveness of knockdown may depend on the type of linker used, and the optimal linker length and composition may vary for different targeted proteins. The linker could also contribute to target specificity. Although a PROTAC may be able to bind to multiple targets, only a subset of potential targets may be presented optimally for efficient ubiquitination and degradation. Therefore, linker selection and PROTAC architecture may present functional selectivity in a nonselective binding ligand when incorporated into a PROTAC molecule [28]. In addition, it was shown that PROTACs have the potential to effectively reduce the levels of specific proteins in various tissues, including solid tumours [28].

PROTAC is a molecule composed of a ligand, usually a small-molecule inhibitor for the POI, and a covalently linked ligand of an E3 ubiquitin ligase (E3). Moreover, the composition of the linker can be used to modify the properties of the PROTAC, such as membrane permeability, aqueous solubility, metabolic stability, and biodistribution. This can help in the development of suitable therapeutic agents and tools. These heterobifunctional molecules simultaneously involve a POI and an E3 ligase, forming a ternary complex. This complex results in the proximity-induced ubiquitination of the POI by the E3 ligase, which subsequently induces proteasome-mediated degradation of the POI itself (Figure 1). The 26S proteasome is responsible for this process, as previously described.

The PROTAC technology is a complementary approach to nucleic acid-based gene knockdown technologies for the targeted reduction of proteins. It can be used to mimic the effects of pharmacological protein inhibition. One of the major advantages of target degradation is the elimination of scaffolding roles. These roles are typically not attenuated by traditional small-molecule inhibitors. A noteworthy attribute of PROTAC technology is that the targeted protein ligands do not necessarily bind to the active sites of the targeted protein, overcoming the limitation of protein inhibitors [29,30], since PROTACs degrade the targeted proteins due to the presence of an E3 ligase. Furthermore, PROTACs possess a substantial advantage over inhibitor molecules in overcoming resistance due to target mutations or overexpression. PROTAC technology has been applied to numerous pathologies and different targets in a number of tumours. In particular, it has been developed in oncology to overcome resistance to targeted therapy in oncogene-addicted NSCLC [31,32,33].

## 3. PROTACs as a Model in EGFR TKIs Pre-Treated Patients: The First Journey

Epidermal growth factor receptor (EGFR) is a transmembrane protein tyrosine kinase. It is a receptor for members of the EGF family regulating cell proliferation, apoptosis, and angiogenesis. The overexpression, mutation, or amplification of the *EGFR* gene increases EGFR activity and contributes to many human cancers, including lung cancers, especially NSCLC. The most frequent activating mutations of *EGFR* in NSCLC are deletions of exon 19 and exon 21 L858R. These mutations occur in the kinase adenosine triphosphate (ATP)-binding domain [34]. As a therapeutic target for NSCLC, *EGFR* has been extensively studied, leading to the discovery of several TKIs. The first-generation TKIs erlotinib and gefitinib have been found to prolong progression-free survival (PFS) compared to chemotherapy in NSCLC patients [35,36]. However, the resistance point mutation p.Thr790Met (T790M) in exon 20 is detected in 50% or more of patients when the disease progresses [37]. Second-generation EGFR inhibitors, like afatinib and dacomitinib, have been developed to overcome resistance [38,39]. Subsequently, third-generation covalent inhibitors have demonstrated greater selectivity for EGFR. Osimertinib has shown superiority in PFS compared to first-generation TKIs [6]. However, several mechanisms have been linked to acquired resistance to third-generation irreversible TKIs, making NSCLC refractory to these inhibitors. The exon 20 C797S point mutation is the dominant on-target resistance mechanism to third-generation inhibitors. In contrast, *MET* amplification and *KRAS* mutations may contribute to the off-target resistance observed in the clinic [40]. Currently, fourth-generation inhibitors are in development and appear to represent a breakthrough against these tertiary mutations. However, there is still an unmet medical need to develop new and more active molecules to overcome the acquired resistance to EGFR. In the field of research, PROTACs represent one of the most promising and effective strategies to overcome acquired resistance to EGFR TKIs (Table 1).

### 3.1. PROTACs Based on First-Generation EGFR TKIs

**Figure 2:**
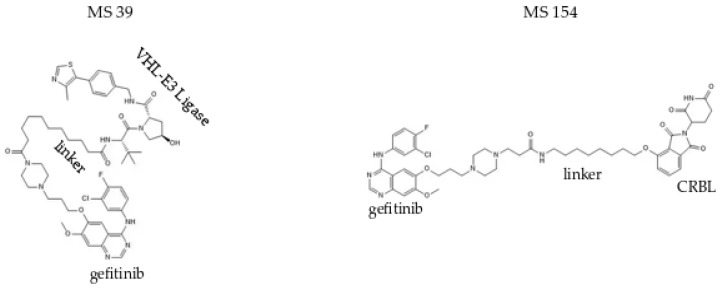
Chemical Structures of MS39 and MS154. MS39 is a selective and effective VHL-recruiting degrader of the mutated EGFR, while MS154 is a CRBN—recruiting degrader of the mutated EGFR [41].

Burslem et al. demonstrated for the first time that PROTACs are capable of inducing the degradation of active receptor tyrosine kinases. Using PROTAC3, derived from the first-generation EGFR inhibitor gefitinib, they also showed that degradation may provide advantages over pure inhibition achieved with TKIs [33]. The ability of this compound to inhibit PD-L1 and IDO1 was subsequently tested. In a study by Wang et al., it was demonstrated that PROTACs significantly reduced PD-L1 and IDO1 levels in NSCLC in vitro and in vivo, making the anti-tumour immune response more effective [52]. Subsequently, a novel gefitinib-based Von Hippel-Lindau (VHL)-recruiting EGFR degrader (compound **6** named MS39) (Figure 2) and a first-in-class gefitinib-based Cereblon (CRBN)-recruiting EGFR degrader (compound 10 named MS154) (Figure 2) were developed. Compounds 6 (MS39) and 10 (MS154) potently induced the degradation of EGFR mutant but not EGFR wild type (wt) in cancer cell lines [41].

Degradation of EGFR by compounds 6 and 10 is mediated by the E3 ubiquitin ligases VHL and CRBN, respectively. In proliferation assays, compounds 6 and 10 inhibited the growth of lung cancer cells. Additionally, while both compounds 6 and 10 were bioavailable in mice, compound 6 was the first EGFR degrader that had sufficient in vivo PK properties, making it suitable for in vivo efficacy studies. Moreover, compound 6 was found to be more active than the previously reported VHL-recruiting EGFR degrader PROTAC3 in inhibiting lung cancer cell proliferation [41]. Yu et al. investigated various E3 ligase-ligands and linkers and discovered two novels, gefitinib-based, highly effective, EGFR PROTAC degraders (compound 31 and compound 72) recruiting VHL and CRBN E3 ligases, respectively. These two degraders showed high binding affinities to both mutant and WT EGFRs but degraded significantly only mutant EGFRs [44].

### 3.2. PROTAC Based on Second-Generation EGFR TKIs

Shi et al. reported the first dacomitinib-based EGFR degraders (compound 13). Compound 13 induced the degradation of EGFR^del19^ in HCC-827 cells, but not other EGFR mutants, wild-type EGFR protein or receptors of the same HER family (HER2 and HER4). Compound 13 was the first EGFR-PROTAC to demonstrate antitumor activity in vivo through ubiquitin-proteasome pathway and inhibit phosphorylation of downstream pathways in vitro and in vivo [47].

### 3.3. PROTAC Based on Experimental EGFR TKIs

Compound F, a purine-containing derivative, was discovered as a highly potent EGFR inhibitor. Compound P3, an EGFR-targeting PROTAC compound F-based, was discovered as a novel degrader capable of effectively inducing both EGFRdel19 and EGFRL858R/T790M degradation in vitro [42]. A new class of EGFR PROTACs degraders based on pomalidomide was developed by Aboelez et al. [43]. All compounds were evaluated for antiproliferative effects in vitro and showed potent inhibitory effects on cell lines. The target compounds exhibited promising activities towards both wild-type and mutant forms of EGFR. Compound 16 was found to be the most potent EGFR inhibitor [43].

### 3.4. PROTACs Based on Third-Generation EGFR TKIs

The third-generation EGFR inhibitors, including rociletinib and osimertinib, have also been used as EGFR binders to develop EGFR PROTACs. Rociletinib (CO-1686) was an irreversible kinase inhibitor that specifically targeted the mutant forms of EGFR, including T790M while exhibiting minimal activity towards the wild-type receptor. A series of EGFR degraders were designed and synthesized by tethering CO-1686 to pomalidomide through several linkers. Promising PROTAC1q could effectively and selectively inhibit the growth of EGFR Del19 and EGFR L858R/T790M cells, but not that of EGFR wt cells. In addition, PROTAC 1q could significantly induce the apoptosis of the cells and arrest the cells in the G0/G1 phase. These results demonstrated that compound 1q could be used for the development of novel EGFR L858R/T790M degradation-based therapy [46]. Zang et al. discovered a potent VHL-recruiting EGFR PROTAC (compounds 14o) that effectively and selectively degraded EGFR L858R/T790M, while showing no effect on the wt protein [48]. Additionally, an osimertinib-derived EGFR degrader (compound 16c) has been described as potently inhibiting EGFR Del19 in vitro [49]. An osimertinib derivative, 7m, with potent anti-tumour activity and high selectivity against EGFR L858R/T790M was developed [53]. Based on a 7m inhibitor, a series of EGFR L858R/T790M degraders recruiting CRBN were designed, synthesized and evaluated. Compound 13a and compound 13b showed anti-tumour effects in vitro and in xenograft nude mice, and they also showed acceptable PK profiles [50].

### 3.5. EGFR PROTACs Based on Pan-ErbB TKI Canertinib

Qu et al. reported the first CRBN-based PROTAC capable of degrading T790M-containing EGFR-TKI-resistant proteins. They synthesized two CRBN-based EGFR PROTACs, SIAIS125 and SIAIS126, based on the EGFR inhibitor canertinib and the cereblon ligand pomalidomide. These two degraders displayed potent and selective antitumor activities in EGFR TKI-resistant lung cancer cells but not in normal cells [45].

### 3.6. EGFR PROTACs Based on ALK TKI Brigatinib

The EGFR C797S mutation is the most common on-target resistance mechanism to osimertinib in patients with advanced NSCLC. Currently, there are no effective treatment options for patients with NSCLC harbouring EGFR C797S mutants. Zhang et al. developed a new series of EGFR PROTACs brigatinib-based targeting Del19/T790M/C797S-mutated EGFR. The representative compound 6h demonstrated that EGFR degradation was mediated by the VHL-associated proteasome pathway, and compound 6h blocked the activation of EGFR downstream signalling [50]. Finally, Du et al. reported the first orally bioavailable EGFR PROTAC, HJM-561, which selectively degraded EGFR C797S-containing triple mutants. HJM-561 was designed to use brigatinib as the warhead of the PROTAC molecule and showed robust antitumor activity in CDX and PDX models that were resistant to osimertinib treatment [51].

## 4. Clinical Applications in Other Actionable Oncogenic Alterations

### EML4::ALK

Anaplastic lymphoma kinase (ALK) is a receptor tyrosine kinase that is aberrantly expressed in several human cancers, including an aggressive subtype of T-cell lymphoma known as ALK-expressing anaplastic large cell lymphoma (ALK+ ALCL), NSCLC and neuroblastoma [54,55]. The leading cause of ALCL is a recurrent t(2;5)(p23;q35) chromosomal rearrangement that gives rise to a fusion protein, NPM::ALK, constituted of 117 N-terminal amino acids of NPM1 protein and the C-terminal part of ALK, including its catalytic domain [56], while the most frequent fusion protein in lung cancer is EML4::ALK found in 5–8% of NSCLC [57,58]. ALK fusions have a clear oncogenic potential since their aberrant tyrosine kinase activity enhances cell proliferation and survival. The most important and well-characterized ALK effectors are the Ras-ERK, JAK3-STAT3 and PI3K-AKT pathways [59]. Crizotinib, a triple MET/ALK/ROS1 inhibitor, was the first clinically available drug to target ALK efficiently. It is approved for treating advanced-stage and metastatic ALK+ NSCLC, and it is under study in clinical trials for other ALK-related diseases. Further ALK TKIs have been developed, including second-generation (alectinib, ceritinib, brigatinib) and third-generation inhibitors (lorlatinib). Currently, ALK-TKIs represent the standard treatment for ALK-positive NSCLC, being proven to be more efficacious compared with chemotherapy [60,61]. However, the use of ALK inhibitors can, over time, induce resistance through well-known mechanisms, including acquired mutation, gene amplification, use of different signalling pathways, and histological transition [62].

PROTAC technology represents one of the most promising strategies to overcome acquired resistance. Several ALK inhibitors, such as ceritinib, brigatinib, alectinib, and gilteritinib, have been used to synthesize PROTACs (Table 2).

PROTACs using ceritinib are TD-004, MI4077, MS4078, TL13-13, TL13-112, and opto-PROTAC-ALK.

TD-004 is a combination of VHL E3 Ligase and ceritinib. It was tested on cell lines with EML4::ALK rearrangement, NPM::ALK fusion, and negative for ALK. Cell lines with NPM::ALK fusion indicated substantial enhancement upon treatment with TD-004, and this effect appeared to continue for a maximum of three days. Unexpectedly, the agent exhibited no effect in the absence of an ALK mutation, while it demonstrated activity on cell lines with EML4::ALK rearrangement. Furthermore, TD-004 was assessed in vivo via intraperitoneal injection for 14 days in xenograft models having EML4::ALK rearrangement with a significant reduction of reduction in tumor volume and absence of side effects [63].

MI4077 (compound 5) and MS4078 (compound 6) are involved in the synthesis of ceritinib and cereblon (CRBN) E3 ligase. They showed activity in vitro against EML4::ALK and NPM::ALK fusion-positive cell lines, leading to reduced levels of fusion proteins. Additionally, they inhibit the ALK autophosphorylation and the activation of the STAT3 pathway. Degradation of NPM::ALK by more than 50% is observed after 5 h, reaching its maximum level at 16 h; whereas degradation of EML4::ALK occurs between 8 and 16 h. The degradation of both fusion proteins is prolonged for at least 24 h [64].

TL13-13 and TL13-112, also known as degraders 9 and 11, are combinations of cereblon E3 ligase with either TAE685 (an ALK inhibitor) or ceritinib. Both compounds demonstrated ALK degradation times in vitro ranging from 4 h to 16 h. They were tested on cell lines expressing EML4::ALK with resistance mutations L1196M, C1156Y, and G1202R, proving effective [65].

Opto-PROTAC is inactive in the dark, but following UVA irradiation, it loses the NVOC group (6-nitroveratroyloxycarbonyl group). Opto-ALK -PROTAC inhibit cell proliferation showing that new modification enables PROTAC to be more effective and precise [70].

PROTAC technology using brigatinib includes SIAIS117, SIAIS164018 and AP-1.

SIAIS117 is capable of inhibiting and degrading ALK protein over 24 h. SIAS117 demonstrated superior growth inhibition in cell lines with G1202R resistance mutation compared to brigatinib [67].

SIAIS164018 consists of brigatinib and cereblon. in vitro experiments demonstrated that it has a greater activity in inhibiting the growth of NPM::ALK fusion cell lines compared to brigatinib alone. It is capable of degrading NPM::ALK and EML4::ALK proteins for over 72 h. Additionally, it degrades the ALK mutation in “G1202R” and the EGFR L858R/T790M. This compound can also arrest the cell cycle in G1 and inhibit the metastatic ability of Calu-1 cell lines [68].

AP-1 is a miniaturized PROTAC, which demonstrated high activity in degrading NPM::ALK and EML4::ALK in vitro, as well as strong antiproliferative effects in vivo [71].

Alectinib-derived PROTACs comprise SIAIS001 and SIAIS091. Both are active in the degradation of NPM::ALK. They were able to effectively bind and degrade both the wild-type and mutant G1202R forms. In addition, the G1/S arrest phase was promoted by SIAIS091 and SIAIS001. Additionally, SIAIS001 demonstrated superior pharmacokinetic properties with good oral availability [69].

To enhance the efficacy of PROTACs, a multi-targeted PROTAC strategy was evaluated called Y-PROTAC. Y-PROTACs are dual PROTACs joined with disulfide bonds. For example, Y5-3 is capable of inhibiting cell growth and its effectiveness is enhanced by glutathione (GSH). Y-PROTACs were shown to degrade ALK with simultaneous CDK4/6 inhibition after the disulfide bond was cleaved by GSH. This ability makes them selective agents targeting cancer cells as normal cells have low levels of GSH [72].

## 5. Other Oncogenic Alterations Besides ALK

HGF is the ligand for the transmembrane receptor c-Met (mesenchymal–epithelial transition). HGF stimulates receptor activation by dimerization, autophosphorylation, and the activation of signalling cascades such as NFkB, STAT, PI3K/AKT, and MAPK upon binding to the receptor at the transmembrane region. A MET exon 14 skipping mutation, protein overexpression, or MET gene amplification can all cause pathological signal activation. MET inhibitors come in three different varieties: Type I (crizotinib, capmatinib, and tepotinib), Type II (cabozantinib and foretinib), and Type III (tivantinib). Capmatinib and tepotinib are FDA and EMA-approved for the treatment of patients with NSCLC harbouring MET exon 14 skipping alterations. Similarly to other oncogenic alterations, resistance to MET inhibitors remains a challenge and MET-PROTACs represent a promising strategy to overcome it.

D10 and D15 are PROTAC compounds (Table 3). They are based on talidomide and tepotinib with linkers of different lengths. Their efficacy has been evaluated in the EBC-1 NSCLC cell line (c-MET overexpression) and the Hs746T gastric cancer cell line (MET exon 14 skipping). D10 and D15 both displayed the ability to inhibit cell migration and invasion, induce apoptosis, and block the cell cycle in G1. Moreover, inhibitory effects on cell proliferation were demonstrated when administered to intraperitoneal EBC-1 xenograft models. Furthermore, D10 and D15 also demonstrated antiproliferative effects in cells harbouring MET Y1230H and D1228N mutations, which were clinically refractory to Tepotinib. At concentrations equal to 10 nmol/L and to 100 nmol/L, D10 and D15 induced 50% and complete degradation of c-MET within 24 h and 12 h, respectively. in vivo results showed that D15 at 20–40 mg/kg reduced c-MET levels, whereas D10 at 40 mg/kg inhibited MET. In the context of enhancing degradation, D15 was observed to be more effective. Furthermore, c-MET degraders at 100 micromol/L exhibited low cytotoxicity to normal cells [73].

The Kirsten Rat Sarcoma viral oncogene homolog (KRAS) is the most common mutated oncogene in various solid tumours, including NSCLC, and it is associated with a poor prognosis. Among KRAS mutations, the most common subtype is G12C, which accounts for 45% of all KRAS mutations in NSCLC and is detected in 13% of cases of lung adenocarcinoma [74]. KRAS has often been referred to as undruggable due to its difficulty in binding to small molecules. Currently, the drugs approved for the treatment of KRAS G12C NSCLC are adagrasib and sotorasib, which are designed to inhibit the target [75,76].

Unlike inhibition, target degradation appears to have a much stronger effect on cell proliferation, as degradation of the molecule eliminates the target and the subsequent downstream pathway at lower concentrations and shorter exposure times. Three PROTACs targeting KRAS have been evaluated.

The first is LC-2, the synthetic association of adagrasib and VHL E3 ligase (Table 3). In vitro studies showed activity on KRAS G12C but not on KRAS G13D cell lines. This demonstrates the specificity towards KRAS G12C. It has rapid action, from 1 h after treatment. Moreover, the degradation of KRAS promotes the inhibition of the development of metastases [77].

Defectinib and VHL E3 ligase were combined to generate D-PROTAC (Table 3), a compound characterized by its ability to alter focal adhesion kinase (FAK), which is crucial for tumour cell proliferation through integrin signal transduction. Defectinib, a FAK inhibitor, demonstrated lower potency compared to D-PROTAC. Indeed, D-PROTAC has been found within in vitro studies capable of reducing both FAK and the phosphorylated form (pFAK) in NSCLC cells, especially those with KRAS mutation, while defectinib was shown to reduce only pFAK. Considerable in vivo validation has been achieved for D-PROTAC’s capacity to minimize tumour cell numbers and prevent tumour invasiveness [78].

KRAS-mutated tumours exhibit high expression of BCL-XL, an antiapoptotic protein. DT2216 is a PROTAC that targets BCL-XL using VHL (Table 3). The combination of sotorasib and DT2216 showed to reduce cell proliferation and promote apoptosis in KRAS G12C, as observed in both in vitro and in vivo studies with mouse xenografts. These effects were achieved without significant impact on blood counts. Currently, a phase 1 study is underway (NCT04886622) [79].

Fibroblast Growth factor receptors are a family of tyrosine kinase receptors expressed on cell membrane (FGFR1, FGFR2, FGFR3, FGFR4) and their pathological activation is implicated in tumour growth, including NSCLC. LG1188 was developed as a pan-FGFR inhibitor, AZD4547 (Table 3). However, it has shown activity in SCLC DMS114 and NSCLC NCI-H1581 cells, selectively degrading FGFR1. Thus, FGFR-PROTACs seem to be more selective than conventional FGFR inhibitors [80].

These PROTACs have shown promising results, but their efficacy needs to be confirmed in further clinical studies.

**Table 3:** PROTACs to target Other Oncogenic Alterations.

Gene	PROTAC	Inhibitor	E3-Ligase	Gene Target	Refs.
*MET*	D10	talidomide	CRBN	Y1230H	[73]
	D15	tepotinib	CRBN	D1228N	[73]
*KRAS*	LC-2	adagrasib	VHL	KRAS G12C	[77]
*FAK*	D-PROTAC	defectinib	VHL	pFAK	[78]
*BCL-XL*	DT2216	sotorasib	VHL	KRAS G12C	[79]
*FGFR*	LG1188	AZD4547	VHL	Pan-FGFR	[80]

MET (Mesenchymal–Epithelial Transition), (KRAS) Kirsten Rat Sarcoma viral oncogene homolog, FAK (focal adhesion kinase), FGFR (Fibroblast Growth factor receptors), PROTAC (Proteolysis Targeting Chimera Agents) PROTAC, VHL (Von Hippel-Lindau), CRBN (Cereblon).

## 6. PROTACs: Strengths and Weaknesses

PROTACs are an innovative technology that provides numerous advantages. By hijacking the ubiquitin-mediated proteolysis system that controls protein levels in eukaryotic cells, PROTACs induce the degradation of target proteins [25]. They work through an “event-driven” mechanism instead of an “occupancy-driven” one [28]. Indeed, their efficacy does not depend on the level of target occupancy, but on kinetic factors such as the expression of the protein being degraded, the efficiency of ubiquitin transfer, and the rate of the target transport by the proteasome. By expanding the set of recruitable E3 ligases, the specificity of PROTACs can be significantly increased [28,81,82]. Even if over 600 E3 ligases are known, the PROTACs design is limited to only a few molecules due to the identification of a small number of ligands to date.

In the context of lung cancer, EGFR, ALK, KRAS, BRAF, Bcl-xl, SHP2 and FAK, E3 ligase ligands, are potential oncogenic targets of PROTACS [83]. Indeed, the current approved drugs work by blocking the active sites of druggable proteins or receptors, but these drugs may become less effective over time through a phenomenon known as drug resistance. Unlike conventional drugs, PROTACs are both selective and catalytic, overcoming resistance and being more cost-effective, especially those used in the treatment of oncogene-addicted NSCLC [84].

Moreover, it is noteworthy that the relative interface between protein and protein complexes is largely hydrophobic and flat. Peptides can modify protein–protein interactions due to their larger contact surface area compared to small molecules [85]. PROTACs can effectively and selectively reduce the expression of various proteins at lower concentrations compared to small molecules that inhibit proteins [33]. This could lead to fewer side effects than TKIs. Furthermore, their mechanism of action may result in a more potent and longer-lasting effect compared to small molecules [33]. Finally, PROTACs offer the potential to broaden treatment targets, including protein kinases, nuclear receptors, regulatory proteins, enzymes, transcription factors, cytokines, and more (Box 1) [66,86,87].

Box 1 **Strengths and weaknesses of PROTACs.**

**Strengths**

- PROTACs induce the degradation of target proteins
- They work through an “event-driven“ mechanism instead of an “occupancy-driven” one
- By expanding the set of recruitable E3 ligases, the specificity of PROTACs can be significantly increased
- Many drivers in lung cancer are potential oncogenic targets of PROTACS
- PROTACs are both selective and catalytic, overcoming resistance to TKIs in oncogene-addicted NSCLC
- PROTACs can effectively and selectively reduce the expression of various proteins at lower concentrations compared to small molecules that inhibit proteins, leading to fewer side effects than TKIs
- They potentially display a more potent and longer-lasting effect compared to small molecules
- PROTACs offer the potential to broaden treatment targets

**Weaknesses**

- The inability of PROTACs to adhere to the well-established drug-like properties associated with oral drugs
- PROTAC molecules can saturate binding sites at high concentrations on the target protein or the E3 ligase without forming the necessary ternary complex for protein degradation
- Resistance can occur to PROTACs due to mutations of target proteins (EGFR, P53) or E3 ligases
- PROTAC technology is not tissue-specific. Thus, administration can induce off-target toxicity and limit combination with other molecules
- PROTACs have low cell permeability thus making it challenging to achieve tissues like the central nervous system and more complicating their oral bioavailability
- Lack of phase 2–3 clinical trials evaluating PROTACs

Abbreviations: PROTACs, proteolysis targeting chimera agents; TKIs, tyrosine kinase inhibitors; NSCLC, non-small cell lung cancer

Despite its strengths, PROTAC technology has several limitations.

The inability of PROTACs to adhere to the well-established drug-like properties associated with oral drugs, namely Lipinski’s Rule of 5 (Ro5), is a major challenge preventing PROTACs from realizing their therapeutic potential [88,89].

PROTAC molecules can saturate binding sites at high concentrations on the target protein or the E3 ligase without forming the necessary ternary complex for protein degradation [90,91,92].

PROTACs can bypass resistance to cancer therapies. Nevertheless, resistance can occur to PROTACs due to mutations of target proteins (EGFR, P53) [93] or E3 ligases [94].

In addition, PROTAC technology is not tissue specific. Thus, administration can induce off-target toxicity and limit combination with other molecules.

In human lung, breast, and colorectal cancer, the most frequently mutated gene is TP53 [95]. Studies have shown that most TP53 mutations occur as missense mutations at specific hotspots within the DNA-binding domain. These include the R175 mutation, as obtained by The Cancer Genome Atlas (TCGA) and the International Agency for Research on Cancer (IARC) analyses. PROTACs have a distinct mechanism for the “destruction” of harmful proteins, yet they cannot distinguish mutant TP53 due to the absence of a druggable active site for small molecules. Recently, Kong L and colleagues demonstrated the inhibition of oncogenic functions of mutant p53 [96]. An aptamer with superior affinity and specificity for p53-R175H, designed through a novel iterative molecular docking approach guided by post-SELEX (systematic ligand evolution by exponential enrichment), has been developed. This aptamer has been used as a ligand for PROTACs, resulting in the development of a selective p53-R175H degrader, named dp53m, which could represent a significant advance in the treatment of cancer [96].

Moreover, PROTACs have low cell permeability thus making it challenging to reach tissues like the central nervous system and further complicating their oral bioavailability.

Finally, the lack of phase 2–3 clinical trials make PROTACs far from being reliable in clinical practise for the treatment of NSCLC (Box 1).

## 7. Conclusions

Lung cancer remains a disease with a dismal prognosis despite the advancement of therapeutics, especially in oncogene-addicted NSCLC. However, there is still an unmet medical need to develop more active molecules to overcome the acquired resistance to the available targeted agents. In this scenario, PROTACs represent an innovative technology with several advantages compared with conventional drugs, mostly being able to induce the degradation of the protein of interest through an “event-driven” mechanism instead of an “occupancy-driven” one. However, many challenges remain as their complexity is not sufficient to ensure effectiveness. Therefore, there is much to achieve before introducing PROTACs into clinical practises, but we have begun to glimpse the light.

## Figures and Tables

**Table 1:** Studies on PROTACs targeting EGFR TK inhibitor resistance.

Gene	PROTAC	Inhibitor	E3-Ligase	mEGFR Target	In Vitro Studies	In Vivo Studies
*EGFR*	PROTAC3	Gefitinib	VHL	Del19–L858R	Yes [33]	No
	MS39			L858R	Yes [41]	Yes [41]
	MS154		CRBN		Yes [41]	Yes [41]
	Compound P3	Compound F	VHL	Del19–L858R/T790M	Yes [42]	No
	Compound 16		CRBN	WT–T790M	Yes [43]	No
	Compound 31	Gefitinib	VHL	Del19–L858R	Yes [44]	No
	Compound 72		CRBN		Yes [44]	No
	SIAIS 125	Canertinib	CRBN	L858R/T790M	Yes [45]	No
	SIAIS 126				Yes [45]	No
	PROTAC1q	Rociletinib	CRBN	Del19–L858R/T790M	Yes [46]	No
	Compound 13	Dacomitinib	VHL	Del19	Yes [47]	Yes [47]
	Compound 14o	Osimertinib	VHL	L858R/T790M	Yes [48]	No
	Compound 16c		CRBN	Del19	Yes [49]	No
	Compound 13a	7m	CRBN	L858R/T790M	Yes [50]	Yes [50]
	Compound 13b				Yes [50]	Yes [50]
	Compound 6h	Brigatinib	VHL	Del19/T790M/C797S	Yes [50]	No
	HJM-561		CRBN	Del19/T790M/C797S L858R/T790M/C797S	Yes [51]	Yes [51]

Abbreviations: PROTAC (Proteolysis Targeting Chimera Agents) PROTAC, VHL (Von Hippel-Lindau), CRBN (Cereblon).

**Table 2:** ALK degraders. Studies on ALK PROTACs synthesized using ALK inhibitors.

Gene	PROTAC	Inhibitor	E3-Ligase	mALK and fALK Target	In Vitro Studies	In Vivo Studies
*ALK*	TD-004	ceritinib	VHL	EML4::ALK NPM::ALK	Yes [63]	Yes [63]
	MI4077		CRBN		Yes [64]	No
	MS4078				Yes [64]	Yes [64]
	TL13-13	TAE685		EML4::ALK L1196M, C1156Y, G1202R	Yes [65]	No
	TL13-112	ceritinib			Yes [65]	No
	dEALK			EML4::ALK	Yes [66]	Yes [66]
	SIAIS117	brigatinib		EML4::ALK G1202R	Yes [67]	No
	SIAIS164018				Yes [68]	Yes [68]
	SIAIS001	alectinib			Yes [69]	Yes [69]
	SIAIS091				Yes [69]	Yes [69]

Abbreviations: PROTAC (Proteolysis Targeting Chimera Agents) PROTAC, VHL (Von Hippel-Lindau), CRBN (Cereblon).
